# The Third Age of Phage

**DOI:** 10.1371/journal.pbio.0030182

**Published:** 2005-05-17

**Authors:** Nicholas H Mann

## Abstract

The third age of phage has begun with the recognition that phages may be key to the great planetary biogeochemical cycles and represent the greatest potential genetic resource in the biosphere

*So, naturalists observe, a flea Has smaller fleas that on him prey; And these have smaller still to bite 'em; And so proceed* ad infinitum.—*Jonathan Swift*

If Jonathan Swift was wrong in envisioning his infinite series of parasites, the “ultimate flea” could be a DNA sequence whose sole biological property is to ensure its own reproduction. But close to the diminutive end of this parasitic spectrum, there are the phages-viruses that infect bacteria. Phages were discovered early in the 20th century, and, at first, interest focussed on the potential of phages as therapeutic tools in the fight against bacterial infectious diseases. The advent of antibiotics was an influential factor in sidelining this ambition. Then for many years the study of phages underpinned the development of modern molecular biology, but that too became passé. The third age of phage has begun only recently with the growing recognition that phages may be major players in the great planetary biogeochemical cycles [1] and also may represent the greatest potential genetic resource in the biosphere.

## Phage Abundance and Diversity

**Figure 1:**
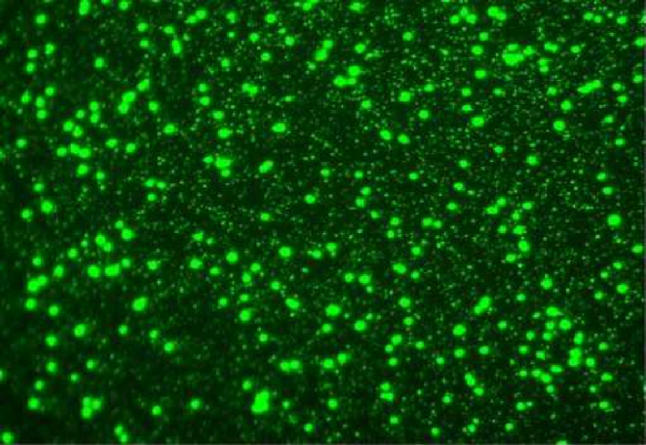
An Image from an Epifluorescence Microscope of Seawater Stained with the Dye SYBR Green to Reveal the Bacterial Cells and Smaller Viral Particles (Image: Jed Fuhrman)

**Figure 2:**
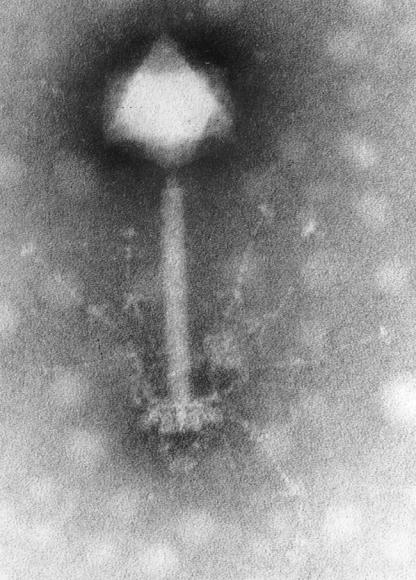
A Transmission Electron Microscope Image of the *Synechococcus* Phage S-PM2 (Image: Hans-Wolfgang Ackermann)

This resurgence of interest in phages may have begun in part with the discovery of the astonishing abundance of viruses in aquatic environments; typically on the order of 107 viruses per millilitre of seawater. Electron microscopy of water samples first suggested the extent of this abundance in the world's oceans [2], which was soon confirmed by faster and more accurate methods for the estimation of virus abundance in seawater based on epifluorescence microscopy (Figure 1) and flow cytometry. It is now widely accepted that phages with a very distinctive morphology, the so-called tailed phages (Figure 2), which dominate the marine virus population, represent the most abundant biological entities on the planet, and total phage abundance in the biosphere has been estimated at 10^30^ or more [3].

It is only very recently, however, through the metagenomic analysis of uncultured marine virus communities, that we have begun gaining an idea of their biodiversity in the marine environment [4]. Approximately 65% of the sequences obtained in a 2002 study did not have homologues in the nucleotide databases, suggesting that marine viral diversity is largely unsampled. Where database homologues were detected, the most common hits were to viruses, including all the major families of tailed phages with double-stranded DNA genomes, and algal viruses. An estimate of the complexity of the virus community structure can be derived by asking how frequently the same sequences are obtained, i.e., how frequently the same individual genome is sampled. On this basis, uncultured phage communities are amongst the most diverse ever analysed, with between 3,000 and 7,000 viral types in a 200-litre sample of seawater [4]. Furthermore, phages can move between very different environments, with very similar phage genes being found in marine, freshwater, sediment, and terrestrial samples [5,6]. This is in keeping with the idea that all tailed phages, regardless of the natural environment of their host, have access to a common gene pool [7].

## Phages Drive Bacterial Diversity

There is also growing evidence that viral activity is a driving force in maintaining genetic diversity amongst the bacterial community and profoundly influences ecosystem functioning [8].The total population of prokaryotes (bacteria and archaea) within the water column of the oceans is collectively known as bacterioplankton and constitutes over 90% of the total biological carbon in the oceans [1]. In the surface layers of the oceans, bacterioplankton populations may be on the order of 10^6^ to 10^7^ cells per millilitre of seawater, and the phage population is typically one order of magnitude larger (reviewed in [9]).

A major component of the total marine bacterial population in the upper illuminated layers of the oceans is marine unicellular cyanobacteria of the genera *Synechococcus* and *Prochlorococcus*. These cyanobacteria make a substantial contribution to the overall productivity of the oceans, both in terms of carbon dioxide fixation and oxygen production. Obviously, phage infection of this extremely important group of organisms will affect the way in which organic carbon is cycled in the oceans. Phages infecting *Synechococcus* strains have been studied since 1993, and the majority of isolates are myoviruses, phages with a double-stranded DNA genome and a long contractile tail (for review see [10]). There is considerable genetic diversity among the *Synechococcus* hosts, and this is reflected in the fact that individual phages only infect certain *Synechococcus* hosts. An analysis of phages infecting *Prochlorococcus* strains revealed a similar pattern of host range variation, with some phages being highly strain-specific and other phages having a broad host range. In fact, some phages were able to infect both *Synechococcus* and *Prochlorococcus* strains [11]. The study by Sullivan et al. [11] yielded one particularly interesting observation: phages that infect strains of *Prochlorococcus* adapted to high light were all viruses with very short tails and very limited host ranges, whilst phages infecting low-light-adapted strains predominantly had broad host ranges and long contractile tails. The evolutionary pressures that have led to this situation are unclear, as are the ecological implications.

## Genome Studies and Insights into Host-Phage Interactions

The study of marine phage genomes is a comparatively recent activity, and only a few genomes have been completed (for review see [12]). However, in this issue of *PLoS Biology*, Sullivan et al. [13] describe the analysis of the genomes of three phages-a podovirus and two myoviruses-capable of infecting *Prochlorococcus* strains. The two myoviruses have large genomes, with one possessing more than 327 potential protein-encoding genes. They are, therefore, genetically complex entities capable of a considerable repertoire of “behaviours”. A key feature of all three genomes is that, in addition to containing typical phage genes, they encode genes specifying proteins whose closest homologues are found in cyanobacteria. Some of these genes are thought to represent “signature” cyanophage genes. Are these genes that have been accidentally acquired from the host and confer no fitness benefit on the phage, or do they indicate important features of how the phage interacts with its host?

Consider, for example, the *psbA* gene that is found in all three phages [14] and has also been found in a *Synechococcus* phage [15]. The D1 protein encoded by *psbA* is a central component of photosystem II, which is responsible for the water-splitting reaction that produces oxygen during photosynthesis. The D1 protein is subject to damage during photosynthesis, and the damaged protein needs to be removed from the reaction centre and replaced by undamaged D1 in a continuous repair cycle in order for photosynthesis to continue. In an uninfected cell the D1 protein is encoded by the cell's genome. However, during infection a common phage strategy is to switch off host gene expression, which could impair photosynthesis and thereby deplete the energy required for viral replication. The provision of a viral D1 protein would permit the repair cycle to continue until the cell lysed to release the phage progeny. Thus, intriguingly, a proportion of the oxygen in the atmosphere may actually be produced by a viral form of photosynthesis.

Another example of cyanophage signature genes are those associated with nutrient acquisition. The central regions of the oceans are nutrient poor, and phosphorus is often likely to be present only in growth-limiting amounts. Both of the *Prochlorococcus* myovirus genomes encode proteins associated with phosphate transport (PstS) or induction in response to phosphate starvation (PhoH) [13].

## The Next Stage for Phage

Thus, the analysis of the genomes of the phages of marine *Synechococus* and *Prochlorococcus* is providing novel insights into phage-host interactions in oceanic low-nutrient environments and considerably extending the paradigms derived from the studies of phages infecting heterotrophic bacteria. However, what genomic studies will not tell us is the proportion of infected cells in the oceans and therefore the true impact of these Swiftian fleas on ocean processes. The answer will require the techniques of molecular ecology, but phage genomics will surely provide the underpinning for such investigations.
